# Exploring Genetic Factors Associated with *Moniezia* spp. Tapeworm Resistance in Central Anatolian Merino Sheep via GWAS Approach

**DOI:** 10.3390/ani15060812

**Published:** 2025-03-12

**Authors:** Yunus Arzik, Mehmet Kizilaslan, Sedat Behrem, Lindsay M. W. Piel, Stephen N. White, Mehmet Ulaş Çınar

**Affiliations:** 1 Department of Animal Science, Faculty of Veterinary Medicine, Aksaray University, 68000 Aksaray, Türkiye; yunus.arzik@aksaray.edu.tr (Y.A.); sedat.behrem@aksaray.edu.tr (S.B.); 2 Department of Animal Science, Faculty of Agriculture, Erciyes University, 38039 Kayseri, Türkiye; mehmet.kizilaslan@tarimorman.gov.tr; 3 Department of Animal and Dairy Sciences, University of Wisconsin-Madison, Madison, WI 53706, USA; 4 USDA-ARS Animal Disease Research 3003 ADBF, Washington State University, Pullman, WA 99164, USA; lindsay.piel@usda.gov; 5 USDA-ARS Poultry Microbiological Safety and Processing Research, US National Poultry Research Center, Athens, GA 30605, USA; stephen.white@usda.gov; 6 Department of Veterinary Microbiology and Pathology, College of Veterinary Medicine, Washington State University, Pullman, WA 99164, USA

**Keywords:** parasite resistance, *Ovis aries*, genome-wide association, *Moniezia* spp., *CD79A* gene, *MAP3K7* gene

## Abstract

Gastrointestinal parasite (GIP) infections pose significant challenges in pasture-based sheep farming, leading to economic losses and welfare concerns. This study aimed to uncover the genetic basis of resistance to *Moniezia* spp. infections in Central Anatolian Merino (CAM) sheep. Genome-Wide Association Analysis (GWAS) was conducted between *Moniezia* spp. egg burden and genomic data from 226 CAM lambs. Thirteen significant Single-Nucleotide Polymorphisms (SNPs) were identified, with five surpassing the genome-wide threshold and eight exceeding the chromosome-wide threshold. Functional annotation revealed associations with genes involved in immune function, notably *CD79A* and *MAP3K7*. *CD79A*, integral to B-cell activation and antibody production, plays a key role in the immune response against parasitic infections. Its interaction with helminth-derived proteins modulates B-cell function, highlighting its potential as a therapeutic target. *MAP3K7*, a central regulator of immune signaling pathways, modulates host responses to helminth infections by influencing NF-κB activity. Additionally, it regulates macrophage function in bacterial infections, showcasing its versatility in mediating immune responses against diverse pathogens. From a practical perspective, the findings of the current research underscore the potential of integrating genomic information into breeding programs to bolster disease resilience in livestock populations for sustainable production purposes. However, further research is needed to elucidate the functional significance of identified SNPs and associated genes. This study underscores the potential of genomic approaches in combating parasitic diseases and promoting sustainable agriculture in sheep production systems.

## Simple Summary

Gastrointestinal parasite (GIP) infections are a major challenge in pasture-based sheep farming, leading to both economic losses and animal welfare concerns. This study aimed to identify genetic factors that contribute to resistance against tapeworm (*Moniezia* spp.) infections in Central Anatolian Merino (CAM) sheep. We conducted genome-wide association studies (GWAS) on 226 CAM lambs, analyzing their genetic data in relation to *Moniezia* spp. egg burden. Thirteen significant genetic markers (SNPs) were identified. Two key immune-related genes, *CD79A* and *MAP3K7*, were linked to parasite resistance. *CD79A* is essential for B-cell activation and antibody production, while *MAP3K7* regulates immune responses, particularly through NF-κB signaling. These findings highlight the potential for using genetic information to improve disease resistance in livestock breeding programs. Further research is needed to understand the role of these genes and to explore host–parasite interactions in more detail.

## 1. Introduction

Gastrointestinal parasite (GIP) infections present notable health challenges in pasture-based farm animal production systems, manifesting in weight loss, anemia, and retrogradation, all of which undermine animal productivity and welfare [1]. These infections impose substantial economic burdens on both farm and national economies, primarily through diminished growth rates and heightened mortality rates among young animals. *Moniezia* spp. infections in sheep are typically of minimal clinical or economic significance. However, heavy infestations in younger animals can lead to symptoms such as abdominal distension, constipation or occasional diarrhea, stunted growth, roughened coat, and anemia. *Moniezia* spp. reside in the small intestines of grazing ruminants. Their life cycle includes soil mites (*Galumna* spp. and *Oribatula* spp.) as intermediate hosts, a stage that can extend up to 16 weeks. During this period, the mites harbor cysticercoids, which are later consumed by grazing ruminants, allowing the parasites to develop into mature tapeworms in the small intestines. [2].

The economic ramifications of *Moniezia* spp. infection extend to direct losses in animal productivity, such as reduced growth rates and lamb deaths, as well as indirect costs associated with intervention strategies, including expenditures on anti-helminthic drugs and veterinary services. According to the studies, even a modest 10% decrease in lamb weight gain during the growth phase can lead to considerable financial losses, estimated at GBP4.40 per lamb [3]. Moreover, in recent years, the use of intensive chemical agents in parasite control has made the sector dependent on anti-parasitic drugs and it has increased year by year [4]. Either the economic burden of using more drugs or the risk of anti-parasitic drug resistance, which parasites have developed more and more every year, has led the industry to use more innovative and sustainable control methods such as the selection of resistant animals. In light of this, addressing the economic losses attributed to parasitic infections is imperative for ensuring the sustainability of sheep production. Consequently, in recent years, efforts in animal breeding have concentrated on creating more resistant herds by integrating complex disease resistance traits into selection indexes, reflecting a disease control strategy embraced by many countries [5,6].

The immune response against gastrointestinal parasites (GIPs) such as *Moniezia* spp. infections in sheep is a complex process influenced by several factors, such as genetics of the host, age, type of parasite, and exposure level and time [7,8]. The immune response involves both innate and adaptive mechanisms. Innate immunity encompasses physical barriers like the mucus layer and smooth muscle contractions in the gastrointestinal system, along with the secretion of bioactive molecules and activation of pattern recognition receptors [9,10]. Additionally, cytotoxic cells like mast cells play a role in defense against parasitic infections [11,12]. The adaptive immune response is primarily orchestrated by antigen-presenting cells, which capture and present antigens to T cells, triggering cytokine secretion and facilitating T cell differentiation [13]. Genes implicated in immune function and host defense mechanisms, such as *CD79A* and *MAP3K7*, play pivotal roles in immune cell signaling, antigen presentation, and cytokine production, suggesting their potential involvement in mediating the host response to tapeworm infections. Therefore, the genetic basis of immune responses in sheep can be partly attributed to variations in these genes, highlighting their importance in disease resistance mechanisms [14,15,16,17]. Genetic factors influencing the synthesis of bioactive molecules and receptors contribute to individual differences in parasite resistance in sheep. Research is ongoing to understand the genetic basis of the immune response against GIP from genome to phenotype [12,14,16,18,19].

Recent advancements in molecular genetics have revolutionized human and animal genetics, particularly with the detailed mapping of the reference genome of the species facilitating the development of Genome-Wide Association Analysis (GWAS) methods. These methods leverage high-density polymorphisms, such as Single-Nucleotide Polymorphisms (SNPs), spread across the entire genome, enabling the understanding of genetic variations and the identification of genomic regions associated with health-related traits, even those with low heritability. GWAS studies have successfully identified genomic regions linked to traits like parasite resistance [20,21,22]. Technological advancements have facilitated the integration of health-related traits, which are often influenced by the collective effects of multiple genes, into genetic improvement programs [23].

The study aimed to uncover the genetic basis of resistance to *Moniezia* spp. infections in sheep and explore the genomic regions underlying this resistance. To achieve this, genome-wide association studies were carried out, between the parasite egg burden in CAM sheep naturally exposed to *Moniezia* spp. infections in pasture and their genome.

## 2. Materials and Methods

### 2.1. Study Population and Phenotype

The study was conducted in the outskirts of Ankara province, Türkiye, characterized by a continental climate with cold, snowy winters and hot, dry summers. The region experiences an annual rainfall of 389 mm, an average temperature of 11.7 °C, and an average altitude of 938 m. Additionally, the region has expansive but predominantly poor and unproductive pastures. A total of 227 CAM lambs, comprising 57 males and 170 females, were selected from three different farms participating in the National Community-based Small Ruminant Breeding Program. A randomization strategy was applied for animal selection on these different farms. This strategy ensured that animals randomly selected from each farm were examined for parasite load and genetic variations. The observed discrepancy in the number of males and females was due to flock management practices, where approximately 25–30% of female animals and about 5% of male animals born that season were retained as breeding stock and remained in the pasture. All animals from these three herds were exposed to the same communal pasture. Born between December 2020 and February 2021, the lambs were weaned at an average age of three months and grazed on pasture during the subsequent summer without additional feed. Selection for the study populations was based on observed growth rates around the mating period (e.g., August–September). Lambs were monitored from birth until fecal sample collection at around six to eight months of age. Environmental factors, including sex, herd, birth type, feeding regime, and location, were recorded for all study animals. The study focused exclusively on *Moniezia* spp. egg types, and other helminth species were not observed in significant numbers due to environmental conditions that limit nematode prevalence. The sample collection was conducted in August, a period identified through preliminary studies as offering the most stable pasture and climatic conditions, ensuring sufficient parasite exposure and minimizing environmental fluctuations. Fecal samples were collected directly from 227 animals at an average age of 6–8 months, with 20–30 g collected per sample to ensure consistency and accuracy in analysis. One animal was excluded from the analysis after genotyping due to failing quality control criteria. It was ensured that at least 60 days had passed since the animals’ last anti-parasite treatment. Blood samples (approximately 6 mL each) were also collected from the jugular veins of the animals during the August sampling. Additionally, the live weights of the lambs were recorded during the collection of fecal samples, and the daily live weight gains were calculated based on birth weights and recorded live weights. Pearson’s correlation was calculated between parasite load and daily live weight gain.

Gastrointestinal parasite (GIP) resistance phenotypes in sheep were determined based on fecal egg counts (FEC) of tapeworms (*Moniezia* spp.), with the number of *Moniezia* spp. eggs counted using the McMaster technique [24] and treated as continuous traits.

### 2.2. Genotyping and Quality Control

The blood samples of 227 sheep were transferred to the Genetics Laboratory of the International Center for Livestock Research and Training (ICLRT) for DNA extraction. DNA extraction was conducted using the Qiacube HT automated device and a commercial kit (Qiagen Blood kit, Hilden, Germany). Quality control checks were performed on the extracted DNA, and samples meeting quality criteria (A260/280>1.8, A260/230>1.5, >70 ng/µL) were transferred to genotyping. Genotyping was carried out using the OvineSNP50 Beadchip Genotyping Array (Illumina Inc., San Diego, CA, USA) with the iScan system according to the user guideline.

Following genotyping, data filtering was conducted to reduce Type-I and Type-II error rates, utilizing quality control criteria established by McCarthy et al. [25], The Wellcome Trust Case Control Consortium [26], and Weale [27]. SNPs with a minor allele frequency (MAF) below 0.05, a call rate under 95%, and those mapped on sex chromosomes were excluded. Additionally, samples with a call rate below 90% and an Identity By State (IBS) value exceeding 95% were removed. SNPs deviating from Hardy–Weinberg Equilibrium (HWE) (i.e., *p*-value = 0.05/number of SNPs) were also excluded.

### 2.3. Statistical Analyses

In instances of missing genotypes, an expected genotype score estimated from the population was utilized for imputation, following the approach outlined by Chen and Abecasis [28]. GWAS analyses were performed using Mixed Linear Models to identify significant SNPs associated with phenotypes. Although incorporating population structure (PS) through principal component (PC) analysis in GWAS models is a common practice, it may not entirely mitigate the risk of erroneous SNP associations. This limitation arises from the inability of population structure analysis to delineate kinship relationships among individuals accurately. Consequently, to address this challenge, we computed the genomic relationship matrix (G) following the method proposed by Astle and Balding [29]. The model incorporated the additive genetic effect of SNPs, with a genomic relationship matrix included to account for covariance between related individuals and population stratification. The following linear model for univariate analysis, utilizing the ‘GenABEL’ package in R (version 1.8.0) [30] to estimate genomic heritability and the SNP effect, was employed.

y = µ+ Xβ + Zu + e

where y represents the vector of individual observations for each blood parameter, µ denotes the population mean for the trait of interest, β signifies the vector of SNP and fixed environmental effects, u indicates the polygenic background effect obtained from MVN (u~0, σ_u_^2^), and e represents the vector of random residual errors obtained from MVN (e~0, σ_e_^2^). X and Z correspond to the design matrices mapping fixed effects and polygenic background effects to each observation, respectively.

Quantile–quantile plots were employed to compare observed test statistics for each SNP to those expected under the null hypothesis of “no association”, aiming to detect any inflation in the test statistics due to systematic biases. Genomic control was applied to p-values, following the method outlined by Devlin and Roden [31], to mitigate higher inflation in test statistics.

Manhattan plots were utilized to visualize the −log10 (*p*-value) of all SNPs relative to their positions on associated chromosomes. Genome-wide and chromosome-wide significance thresholds, determined using Bonferroni correction, were applied to minimize Type-I Error rates arising from multiple testing of SNPs. Accordingly, the genome-wide significance threshold was set at 1.11 × 10^−6^ (0.05/44,871), and the chromosome-wide significance threshold was set at 2.89 × 10^−5^ ((0.05/44,871) × 26).

### 2.4. Functional Gene Annotation

Positional information for significant SNPs and nearby genes was obtained from NCBI Genome Data Viewer using the Oar_v4.0 genome assembly [32]. Genes overlapping with SNPs were considered as candidates, and if the significant SNP was not located within a gene, a scan within ±25 kbp distance from the SNP was conducted to identify candidate genes. Functional annotation on identified candidate genes was acquired using DAVID Bioinformatics Resources 2021 [33]. In cases where annotation in the sheep genome reference was insufficient, orthology between species was utilized, and annotations from cattle, goats, mice, and humans were employed. Furthermore, the biological processes involving the genes of significant SNPs were represented using their Gene Ontology (GO) terms, which can be further explored on the QuickGo website [34].

## 3. Results

The outliers in the fecal egg count for *Moniezia* spp. were detected and eliminated from the dataset, yielding a mean egg count of 646 ± 176 per gram. Comprehensive details regarding the phenotype data are provided in Table S1. Initially, the raw genotype dataset consisted of 49,355 SNPs for 227 animals. Following quality control procedures applied to the genotypic data, 44,871 SNPs and 226 animals were retained for further analysis.

Prior to the GWAS analysis, a linear mixed model was employed to assess the effects of fixed factors. Results indicated that sex and herd significantly influenced egg count (see Table S1). Consequently, these factors were incorporated into the GWAS models. Notably, herd and age were found to exert statistically significant effects, warranting their inclusion in subsequent analyses. Genomic heritability estimates were found to be 0.14 for the trait. Additionally, the live weights of the lambs were recorded during the collection of fecal samples, and the daily live weights were calculated based on birth weights and recorded live weights. Pearson’s correlation was calculated between parasite load (*Moniezia* spp. egg count) and daily live weight gain. Pearson’s correlation between parasite load and daily live weight gain was negatively correlated (r = −0.15, *p*-value < 0.05).

The Q-Q plots of observed test statistics for each SNP, comparing them with those expected under the null hypothesis, were generated (Figure S1). The Q-Q plots, along with the estimated inflation factor lambda (λ), were obtained (0.99) for the phenotype, with genomic control applied to normalize the data. These plots confirmed the appropriateness of our model, and the adequacy of genomic control applied.

**Figure 1:**
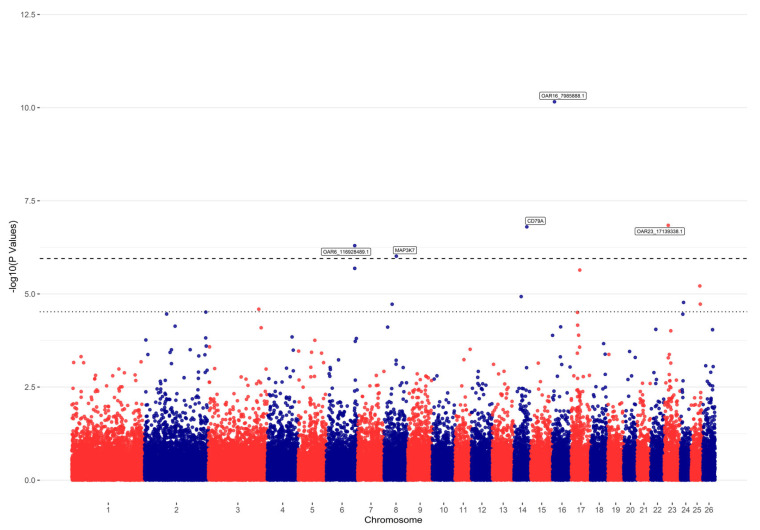
Manhattan plots showing −log10 (*p*-values) of association between SNPs and phenotype. Each dot represents the result from the test association for a single SNP. The upper horizontal dashed line shows a genome-wide threshold with −log10 (1 × 10^−6^) and the lower dashed line shows a chromosome-wide threshold with −log10 (2 × 10^−5^).

GWAS identified several significant SNPs associated with tapeworm resistance. Corrected *p*-values for each trait were derived, and Manhattan plots illustrating these results are provided in Figure 1. Genome- and chromosome-wide significance thresholds are indicated by dashed lines, with 5 genome-wide significant SNPs and 9 chromosome-wide significant SNPs identified in the GWAS for the egg count of CAM lambs. Further details on significant SNPs can be found in Table 1.

In terms of functional annotation, based on positional information obtained from NCBI Genome Data Viewer using the Oar_v4.0 assembly, 8 of these SNPs were directly associated with specific genes. Among these 8 SNPs, 5 were found within 6 distinct genes, while the remaining 3 were associated with 6 different genes within a ±25 kbp distance. These findings provide insight into potential genetic mechanisms underlying *Moniezia* spp. resistance in sheep.

The identified genes include *CD79A*, *ARHGEF1*, *LYPD4*, *RPS19*, *DMRTC2*, *FAM193A*, *MAP3K7*, *SH3BP2*, *FAT4*, *CDH8*, *QRSL1*, *RTN4IP1*, and *ABCC9*. The roles of these genes suggest a range of biological processes potentially involved in immune response and resistance to *Moniezia* spp. infection. For instance, *CD79A* is involved in the initiation of immune responses, and *MAP3K7* plays a role in inflammatory responses.

## 4. Discussion

Gastrointestinal parasites cause significant weight loss and stunted growth in sheep. Genomic heritability estimates for fecal egg count (FEC) of *Moniezia* spp. in CAM sheep yielded a relatively moderate value of 0.14. Conversely, a study on the Akkaraman sheep breed reported a higher genomic heritability estimate of 0.30 for the FEC of *Moniezia* spp. in lambs [22]. The disparity in genomic heritability estimates observed between these two breeds may suggest genetic differences in parasite resistance. The findings of this study shed light on the genetic basis of resistance to *Moniezia* spp. infections in sheep and provide insights into the genetic mechanisms underlying this resistance. By conducting genome-wide association studies (GWASs) between the parasite egg burden in CAM sheep and their genome, we identified 13 significant SNPs, with 5 surpassing the genome-wide threshold and 8 exceeding the chromosome-wide threshold.

The strongest association was observed with OAR16_7985888.1 (*rs413439386*) on chromosome 16 (Table 1). In official genome annotations, this SNP is located in a gene desert. The closest genes are ENSOARG00020036333 and ENSOARG00020038705, located on either side of *rs413439386* at distances of approximately 150–200 kbp. ENSOARG00020036333 is a protein-coding gene with homology to reverse transcriptase. ENSOARG00020038705 encodes a long noncoding RNA. If this is the true extent of local genes, then rs413439386 or a nearby variant could function through regulatory effects, and further work will be required to define such function. Interestingly, Genscan predicts a gene (Chr16.178) in reverse orientation with exon 3 overlapping the position of *rs413439386* on Oar_v4.0, as shown on the UCSC genome browser [35]. If this gene prediction is correct, then *rs413439386* encodes a charged R111Q substitution. More work will be required to determine if this gene is expressed in certain contexts, and if so, how it functions in the context of *Moniezia* spp. infection.

In the current study, the *CD79A* and *MAP3K7* genes located on chromosomes OAR 14 and 8, respectively, were found to be associated with the fecal egg count of *Moniezia* spp. In Scottish Blackface sheep, a wide QTL span (0–121 cM) covering the *BMS833* and *ILSTS02* QTLs, identified through microsatellite marker-based association analyses, was associated with *Nematodirus* egg count [36]. Additionally, in a study conducted on Akkaraman sheep, the *ATRNL1* gene on chromosome 22 was linked to *Moniezia* spp. egg count [22]. The utilization of animals from different breeds in the current and previous studies provides the most reasonable explanation for the associations of different chromosomes and genes observed in these studies.

The *CD79A* gene, encoding the Ig-alpha protein of the B-cell antigen receptor complex, emerges as a key player in the immune response against parasitic infections in sheep [37]. This gene is integral to the functionality of the B lymphocyte antigen receptor complex, which includes the antigen-specific component surface immunoglobulin (Ig). The surface Ig, in association with Ig-alpha and Ig-beta proteins, forms the B-cell antigen receptor essential for B-cell activation and antibody production. In the context of parasite resistance, *CD79A*’s involvement in adaptive immune response (GO:0002250), B cell differentiation (GO:0030183), B cell proliferation (GO:0042100), B cell receptor signaling pathway (GO:0050853), B cell receptor complex (GO:0019815), IgM B cell receptor complex (GO:0071755), and B cell receptor signaling pathway (K06506) underscores its significance in mounting an effective immune response against invading pathogens such as *Moniezia* spp.

The role of *CD79A* in parasite–host interactions has been elucidated through various studies across different helminth species. Na-ASP-2, a secreted venom allergen-like protein from *N. americanus*, has been elucidated to bind to *CD79A*, thereby modulating B-cell responses [38]. Through its interaction with CD79A, Na-ASP-2 downregulates B-cell receptor signaling pathways (GO:0050853), resulting in altered gene expression profiles and impaired B-cell function [12,18]. This interaction highlights the sophisticated mechanisms employed by parasites to evade host immune responses and establish successful infections.

Moreover, Ryan et al. [15] showed that the binding of Na-ASP-2 to *CD79A* not only affects B-cell function but also influences other immune processes, such as leukocyte transendothelial migration (GO:0002686). The downregulation of key molecules involved in leukocyte migration pathways further underscores the immunomodulatory effects of CD79A-binding proteins secreted by parasitic helminths [16,39]. This highlights the multifaceted role of *CD79A* in orchestrating immune responses against parasitic infections and the intricate interplay between host and pathogen.

Furthermore, the interaction between *CD79A* and helminth-derived proteins presents potential implications for vaccine development and therapeutic interventions. Understanding the molecular mechanisms underlying CD79A-mediated immune modulation provides valuable insights into host–parasite interactions and may pave the way for the development of novel strategies for controlling parasitic infections. Overall, the evidence gathered from the literature underscores the significance of *CD79A* in parasite resistance and highlights its potential as a target for therapeutic intervention in combating parasitic diseases in sheep and other susceptible hosts.

The *MAP3K7* gene, also known as Transforming growth factor b-activated kinase 1 (*TAK1*), emerges as a crucial regulator of immune responses against parasitic infections in sheep. This gene encodes a serine/threonine protein kinase that mediates signaling transduction induced by various stimuli, including cytokines and environmental stresses [40]. *MAP3K7* plays a central role in the activation of nuclear factor kappa B (NF-κB) and mitogen-activated protein kinase (MAPK) signaling pathways, thereby controlling a multitude of cellular functions such as transcription regulation, apoptosis, and inflammatory responses.

Studies investigating the role of *MAP3K7* in parasite resistance have highlighted its significance in involving immune responses against helminth infections. In chronic gastrointestinal helminth burdens, MAP3K7-binding protein 2 (TAB 2), a downstream effector of MAP3K7 signaling, was positively correlated with parasite burden, indicating its involvement in modulating host responses to parasitic infections [19]. During *Schistosoma mansoni* infection, adult parasites secrete miRNA-containing extracellular vesicles that target MAP3K7 in T helper cells, leading to downmodulation of NF-κB activity and subsequent suppression of the Th2 immune response [14,17]. This mechanism elucidates how parasites evade host immune surveillance by manipulating key signaling pathways involved in immune cell differentiation and function.

The rewiring of MAPK signaling and activation of MAP3K7/TAK1 kinase has been associated with the induction of macrophage function in response to bacterial infections. The PMA-induced transition of monocytes to macrophages resulted in the upregulation of *MAP3K7*, highlighting its role as a central signaling hub in bacterial killing and chemokine production [40]. These findings underscore the versatility of *MAP3K7* in mediating immune responses against a diverse range of pathogens, including both bacterial and parasitic infections. Understanding the intricate signaling networks involving *MAP3K7* provides valuable insights into host–parasite interactions and may pave the way for the development of novel therapeutic strategies targeting key signaling molecules to enhance parasite resistance in sheep and other susceptible hosts.

While the results of this study provide valuable insights into the genetic factors influencing resistance within the Central Anatolian Merino sheep breed, we recognize that broader applications to other sheep breeds in Türkiye or globally may require further investigation. The limited number of farms sampled may restrict the universality of the findings; however, within the context of the specific environmental conditions of the study area, the results demonstrate a level of generalizability concerning the genetic basis of resistance to tapeworm infections [41,42]. Therefore, this study serves as a foundational step, highlighting the need for additional research on diverse populations and breeds to fully understand genetic resistance to parasites across different environments. Expanding future studies to include a wider range of breeds and populations would enhance the understanding of these genetic mechanisms and their applicability in breeding programs aimed at improving parasite resistance.

By identifying genomic regions and the genetic mechanisms associated with parasite resistance traits, such as *Moniezia* spp. egg count, breeders can implement targeted selection strategies to improve resilience to GIP infections. This approach aligns with the broader goal of sustainable agriculture and animal husbandry by reducing the reliance on chemical interventions and promoting genetic resilience to parasitic diseases. However, it is essential to acknowledge certain limitations of our study. While GWAS provides valuable insights into the genetic basis of complex traits, including parasite resistance, further research is needed to elucidate the functional significance of the identified SNPs and associated genes. Functional studies, such as gene expression analyses and in vitro assays, are warranted to validate the roles of candidate genes in the immune response to *Moniezia* spp. infections.

## 5. Conclusions and Recommendations

The current study underscores the genetic heterogeneity underlying resistance to *Moniezia* spp. infections in sheep, highlighting the potential of genomic approaches to enhance disease resilience in livestock breeding programs. Our findings, including the identification of significant SNPs associated with *Moniezia* spp. resistance, such as those on chromosomes 8 and 14, provide a valuable foundation for targeted breeding strategies. Notably, the *CD79A* gene, involved in immune responses, and the *MAP3K7* gene, a crucial regulator of immune signaling, were linked to fecal egg counts, suggesting their important roles in modulating resistance to *Moniezia* spp. infections. The significant SNP *rs413439386* on chromosome 16, although located in a gene desert, potentially influences nearby genes involved in immune regulation. While GWAS offers valuable insights into the genetic basis of complex traits like parasite resistance, further functional studies are necessary. Gene expression analyses and in vitro assays will be crucial for validating the roles of the identified genes in the immune response to *Moniezia* spp. infections. Additionally, the genetic architecture of parasite resistance is influenced by host genetics, environmental conditions, and parasite diversity. Future research with larger sample sizes, longitudinal data, and multi-omics approaches will provide a more comprehensive understanding of the host–parasite interactions in livestock populations. Given the significant role of B-cells in the immune response to parasitic infections, we recommend exploring the potential of other analyses using serum from animals exposed to *Moniezia* spp. proteins. Identifying specific antibodies involved in resistance could lead to the development of genetic selection and vaccination strategies. These approaches have the potential to enhance our ability to develop effective methods for controlling *Moniezia* spp. infections in sheep and can improve overall resilience in livestock breeding programs by incorporating parasite resistance into selection programs using index methods, while considering its genetic correlations with other performance and adaptation traits.

## Figures and Tables

**Table 1:** Significant SNPs associated with the tapeworms’ fecal egg count.

SNP Name	rs id	Chr.	Position (bp) ^a^	*p*-Value	Significance Level	Associated Genes	
						Name	Distance (bp)
OAR16_7985888.1	rs413439386	16	7482199	6.93 × 10^−11^	*GW*	*-*	-
OAR23_17139338.1	rs403168292	23	15978964	1.44 × 10^−7^	*GW*	*-*	-
s21643.1	rs399780906	14	50299438	1.58 × 10^−7^	*GW*	*CD79A* *ARHGEF1* *LYPD4* *RPS19* *DMRTC2*	Within ~4 kb ~25 kb ~9 kb ~21 kb
OAR6_116928489.1	rs424147325	6	115312997	5.07 × 10^−7^	*GW*	*FAM193A*	Within
S04655.1	rs409459191	8	46831736	9.70 × 10^−7^	*GW*	*MAP3K7*	~3 kb
S65350.1	rs422791391	6	115244531	2.06 × 10^−6^	*CW*	*SH3BP2*	~20 kb
OAR17_35112051.1	rs4039744896	17	32126658	2.29 × 10^−6^	*CW*	*FAT4*	Within
OAR25_35637536.1	rs398917787	25	34107540	6.10 × 10^−6^	*CW*	*-*	-
OAR14_29936907.1	rs408792120	14	28689202	1.18 × 10^−5^	*CW*	*CDH8*	Within
S50538.1	rs425776415	24	11986747	1.69 × 10^−5^	*CW*	*-*	-
OAR25_37746412.1	rs430437733	25	35851406	1.87 × 10^−5^	*CW*	*-*	-
OAR8_33063143.1	rs426900187	8	30374437	1.89 × 10^−5^	*CW*	*QRSL1* *RTN4IP1*	Within Within
OAR3_207847578_X.1	rs413235350	3	192922322	2.58 × 10^−5^	*CW*	*ABCC9*	Within

^a^ SNP position based on Oar_v4.0 assembly.

## Data Availability

The original contributions presented in the study are included in the article; further inquiries can be directed at the corresponding author.

## Supplementary Materials

The following supporting information can be downloaded at: https://www.mdpi.com/article/10.3390/ani15060812/s1, Figure S1: Quantile-quantile plot compared the observed distribution of −log (*p*-values) to the expected values under the null hypothesis. Table S1: Descriptive statistics and significant fixed effects accounted for during association analyses.
