# Supplementary material for: Exploring Genetic Factors Associated with Moniezia spp. Tapeworm Resistance in Central Anatolian Merino Sheep via GWAS Approach
Source: Animals (Basel). 2025 Mar 12;15(6):812. doi: 10.3390/ani15060812 (PMC11939720; doi:10.3390/ani15060812)
Supplement: Supplementary file 1 [file animals-15-00812-s001.zip › animals-3393859-supplementary.pdf]

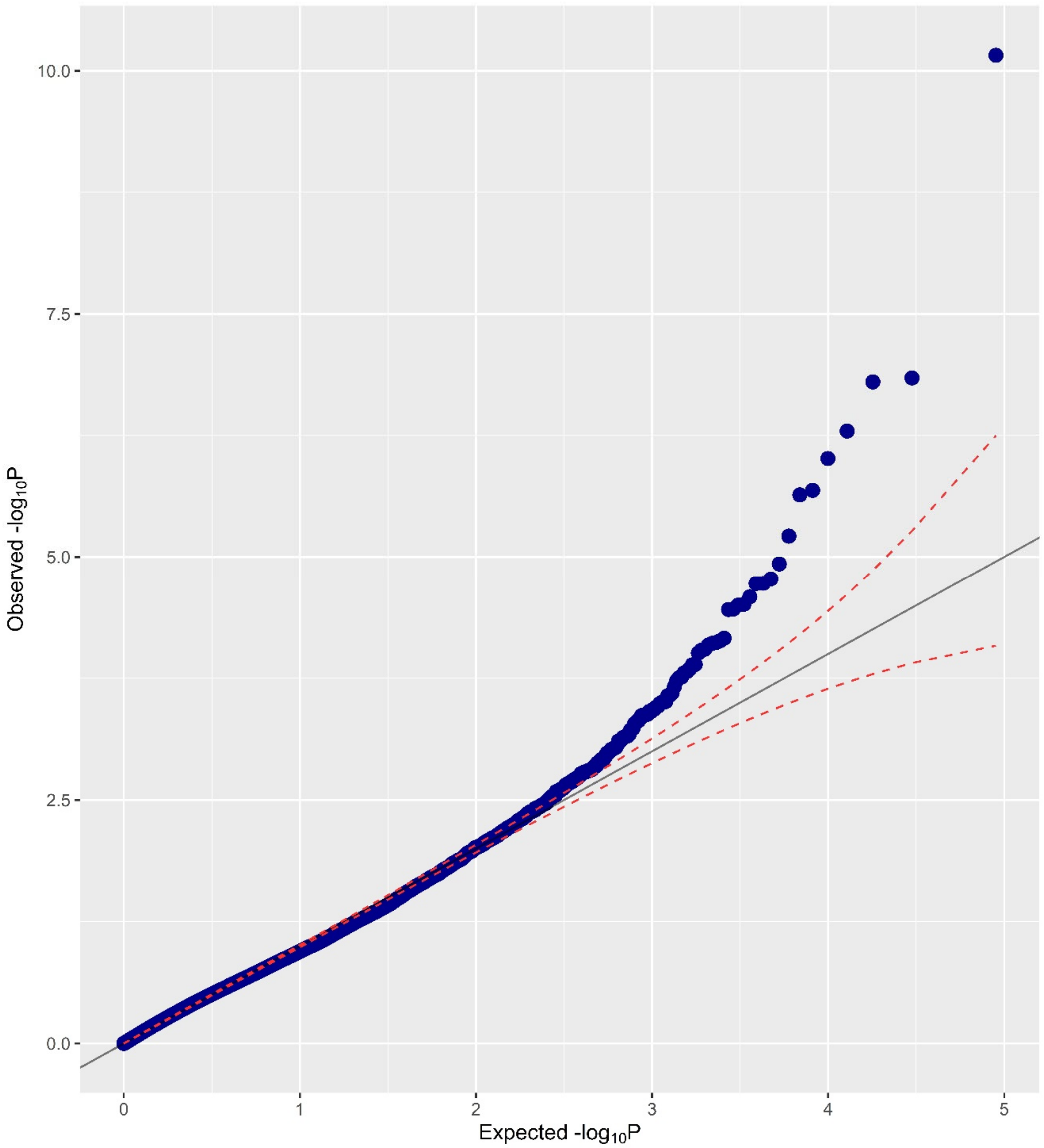

Figure S1: Quantile-quantile plot compared the observed distribution of –log (p-values) to the expected values under the null hypothesis.

Table S1. Descriptive statistics and significant fixed effects accounted for during association analyses.

Traits	N	Mean	SE Mean	Min[a]	Max[b]	SD[c]	Significance level[d]
**Fecal egg count**	226	646	176	0	33,939	2,661	-
**Sex**							*
**Male**	57	964	205	0	33,939	2,674	
**Female**	169	540	347	0	15,960	2,621	
**Herd**							***
**1**	139	796	258	0	33,939	3,353	
**2**	41	87	52	0	176	332	
**3**	46	758	371	0	15,970	2,516	

[a]Min: minimum, [b] Max: maximum, [c]SD: standard deviation, [d] *p-value < 0.05; **p-value < 0.01; ***p-value < 0.001;.
